# Does Workplace Bullying Produce Employee Voice and Physical Health Issues? Testing the Mediating Role of Emotional Exhaustion

**DOI:** 10.3389/fpsyg.2021.610944

**Published:** 2021-02-09

**Authors:** Huai-Liang Liang

**Affiliations:** Department of International Business Management, Dayeh University, Changhua, Taiwan

**Keywords:** workplace bullying, harassment among peers/peer bullying, employee voice, emotional exhaustion, physical health

## Abstract

Workplace bullying is a reality in organizations. Employees’ experiences of workplace bullying can produce their voice that intends to challenge the *status quo* at work and can damage their physical health. This study examines the effects of workplace bullying on employee voice and physical health issues and considers individuals’ emotional reactions as a critical mechanism operating between workplace bullying and its consequences in workplace situations. Emotional exhaustion mediates the influence of workplace bullying on employee voice and damaged health. Data for 694 employees from a large Taiwanese retail organization revealed that workplace bullying relates to its outcomes at work. The findings of this study show that emotional exhaustion is a critical mechanism between workplace bullying and its consequences, i.e., employee voice and health issues. A time-lag study design is applied to reduce common method bias.

## Introduction

Workplace bullying is a serious problem in work life that decreases the degree of job attitude (e.g., work engagement; Einarsen et al., 2016). The victims of workplace bullying experience systematic actions of social exclusion (Leymann, 1990) and aggression from employers, colleagues, and/or subordinates (Nielsen and Einarsen, 2012). Prior studies have indicated that workplace bullying produces extreme psychological issues (e.g., strain and mental problems) and physical reactions (e.g., violence) among bullied victims (Tuckey and Neall, 2014; Kwan et al., 2016). A number of studies have addressed the effects of workplace bullying on victims’ negative behaviors, such as absenteeism (Magee et al., 2017), turnover intention (Samnani and Singh, 2012), lower work engagement (Einarsen et al., 2016; Park and Ono, 2017), and job dissatisfaction (Nielsen and Einarsen, 2012). Employers should aim at the crucial workplace issue both in preventing bullying from happening and in decreasing its possible negative consequences. However, little research has aimed at the positive behaviors of victims, who may express their opinions and suggestions to the organization to avoid lasting workplace bullying.

Affective events theory (AET; Weiss and Cropanzano, 1996) is that a work event should be conceptualized as a valuable statement regarding the employee’s emotional reaction. AET denotes the procedure in which individuals aim to affect employee responses in the workplace to lead to their behavioral consequences at work (Park and Ono, 2017). Given the bullying process, the victims who experience a negative bullying environment are more likely to be recklessly determined to respond by using voice to alter the bullying situation. When employees experience threats to their personal social condition and physical pressure in the workplace, they may seek to reinforce their status in an organization (Van Dyne and Ang, 1998). Kwan et al. (2016) reported that employees can voice their bullying problems in a safe organizational climate because this type of climate implies that individuals are willing to address threats on behalf of others. At the same time, organizations should also be required to deal with workplace bullying effectively, thereby diminishing the damage suffered from bullying when it occurs. The first purpose of this study is to examine the bullied victims’ use of voice, which is an active behavior adopted to alter the bullying situation, as a response to workplace bullying.

Although prior studies have investigated that workplace bullying relates to mental health issues, such as loss of security, anxiety, and depression (Bernotaite and Malinauskiene, 2017; Finstad et al., 2019; Lopes et al., 2020), in addition, workplace bullying can directly or indirectly damage health (e.g., sleep problems and suicide; Strandmark and Hallberg, 2007; Samnani and Singh, 2012). The negative effects of workplace bullying on health strongly influence individuals’ lives (Kwan et al., 2016); approximately $64 billion is spent annually by organizations and society to cope with bullying (Query and Hanley, 2010). A prior research found that 75.6% of victims bullied in the workplace reported that their health was negatively influenced by their experience (Park and Ono, 2017). Therefore, the second purpose of this study is to understand how workplace bullying may influence victims’ physical health issues.

This study attempts to employ AET (Weiss and Cropanzano, 1996) to examine the negative emotional reaction produced by the targets of bullying over time and whether this results in physical issues (e.g., Nielsen and Einarsen, 2012). Workplace bullying is recognized as a serious reality in organizations (Park and Ono, 2017). When the bullied victim is unable to cope with workplace bullying, the degree of the emotional response may lead to constant exhaustion. Emotional exhaustion is defined as “a chronic state of physical and emotional depletion that results from excessive job demands and continuous hassles” (Wright and Cropanzano, 1998, p. 486). The more emotional exhaustion the victim sustains, the more likely it is that the victim will experience adverse effects on physical health (Bond et al., 2010; Nielsen and Einarsen, 2012; Kwan et al., 2016). Therefore, this study suggests that workplace bullying may produce emotional exhaustion, a negative emotion that leads employees to express fewer opinions aiming to improve the workplace bullying environment and results in the victim’s negative physical health.

In summary, this study advances the understanding of workplace bullying by (a) directly investigating the effects of workplace bullying on voice behavior, in which the victim may recklessly use voice to alter the bullying situation; (b) examining the relationship between workplace bullying and health issues; and (c) proposing the role of emotional exhaustion as a critical mechanism between workplace bullying and its outcomes (i.e., voice behavior and physical issues) by drawing on AET.

## Theory and Hypotheses

### Affective Events Theory

In the last two decades, studies have used AET to examine the reasons for and outcomes from individual emotions in the workplace (Weiss and Cropanzano, 1996; Ashkanasy and Daus, 2002; Lange et al., 2011). AET emphasizes the processes behind employee affective responses in the workplace and focuses entirely on the processes of individual judgment (Weiss and Cropanzano, 1996). Workplace events may produce affective responses among employees, leading to attitudinal and behavioral consequences at work. For example, Park and Ono (2017) demonstrated that workplace bullying (e.g., a negative workplace) can cause a reassessment of psychosocial safety in the work environment, which influences employees’ work engagement. Psychological and emotional experiences may trigger victims of workplace bullying and thereby produce negative effects. Therefore, this study aims at investigating the affective emotional process in the relationship between workplace bullying and its consequences.

### Workplace Bullying and Voice Behavior

Workplace bullying is defined as systematically harassing, insulting, and socially ignoring someone and harmfully disrupting someone’s work assignments (Einarsen, 2000). Prior studies have reported that workplace bullying includes repetitive actions of harassment, including vocal violence, a power inequity between offenders and targets, and repeated aggressive and negative behaviors (Einarsen, 2000; Samnani and Singh, 2012; Park and Ono, 2017). Although there are many definitions of bullying behavior, workplace bullying mainly includes verbal hostility, social isolation, the spreading of rumors, attacks regarding personal behavior, and physical threats (Leymann, 1990; Zapf et al., 1996; Nielsen and Einarsen, 2012). Workplace bullying can represent violent behavior from supervisors, subordinates, and colleagues (Einarsen, 2000). Therefore, the concept of bullying involves routine aggression and violence directed toward people by a group or by one person (Galanaki and Papalexandris, 2013). Furthermore, prior studies have reported that workplace bullying as a prolonged process develops into chronic and cognitive activation (Eriksen and Ursin, 2004; Nielsen and Einarsen, 2012). The main idea of this study is that workplace bullying is a form of unsolved divergence and a process in which a period of continued bullying experience leads to cognitive activation. During the bullying process, therefore, the bullied victims are more likely to use a helpful change-oriented communication to alter the bullying situation. They may use voice more frequently because managers can attend the content of voice to address workplace bullying issues.

Employee voice is seen as a behavior rather than as a perception (Weiss and Morrison, 2019). LePine and Van Dyne (2001) defined voice as a “constructive change-oriented communication intended to improve the situation” (p. 326) and as voluntarily originating beneficial changes in organization (Grant et al., 2009). Voice behavior involves bringing potential problems to employer attention and making valuable suggestions to organizations (Ng and Feldman, 2012). Employee voice can enhance an individual’s status in an organization and impact interpersonal relationships (Van Dyne and Ang, 1998) because it implies an individual’s willingness to address threats on behalf of others. The most important way in which individuals can engage in voice is by constructively challenging the *status quo* by presenting opinions or raising concerns about workplace issues (Weiss and Morrison, 2019). Voice expressions are likely to propose ways to resolve problems with work processes and situations in which individuals experience unfair treatment in the workplace (Maynes and Podsakoff, 2014). Therefore, bullied victims may respond effectively to voice because change proposals that address workplace bullying may be seen as preventing serious psychological and physical harm and suggesting improvements to standard social relationships in the organization. This reasoning leads to the following hypothesis.

Hypothesis 1: Workplace bullying is positively related to employee voice.

### Workplace Bullying and Health Issues

Empirical evidence has shown that when bullied victims are unable to deal with bullying in the workplace, a negative physical condition occurs to some degree (Eriksen and Ursin, 2004). Prior studies have stated that all victims experience physical issues, such as shaking, sweating, or sleeplessness, when bullying occurs (Adams, 1992; Hoel et al., 2004; Kwan et al., 2016). According to a meta-analytic review of 66 samples (Nielsen and Einarsen, 2012), workplace bullying influences physical and mental issues over time (e.g., physical health problems and post-traumatic stress). Another serious problem is that bullied targets generate harmful behaviors when they are unable to cope with the bullying situation. Based on AET, it has been suggested that the failure to address experiences of workplace bullying causes negative emotional, lasting mental, and damaging physical reactions (Nielsen and Einarsen, 2012). The investigations leading to the above results capture victims’ own experience of damaging health from the bullying event. Therefore, this study hypothesizes that workplace bullying may produce physical health issues.

Hypothesis 2: Workplace bullying is negatively related to physical health.

### Workplace Bullying and Emotional Exhaustion

This study focuses on the emotional exhaustion dimension of burnout, which is highly indicative of distress and mental fatigue in an emotionally severe work environment (Leiter and Schaufeli, 1996). The reasons for investigating emotional exhaustion are as follows. First, emotional exhaustion is essential to the experience of burnout and the core symptom of job burnout (Lee and Ashforth, 1996; Halbesleben and Bowler, 2007). Second, previous studies have investigated relationships between burnout components, antecedents, and consequences (Green et al., 1991; Lee and Ashforth, 1996; Halbesleben and Bowler, 2007). Investigation of emotional exhaustion has revealed that the most reliable components are its relationship with variables such as job demands and job performance (Demerouti et al., 2001; Schaufeli and Bakker, 2004; Halbesleben and Bowler, 2007).

In theory, workplace events could affect positive or negative emotional reactions, resulting in an increase or decrease in inherent work motivation and individual behaviors over time (Weiss and Cropanzano, 1996). Indeed, workplace bullying as a negative workplace event reflects an inability to deal well with the situation, which means that the level of emotional reaction may be sustained. Workplace bullying reflects the extent to which victims experience long-lasting uncomfortable hostility and aggression. Prior studies have found that a self-reported bullying situation increases the levels of stress and depression (Mikkelsen and Einarsen, 2002; Hoel et al., 2004; Nielsen and Einarsen, 2012). In addition to these stress reactions, it has also been suggested that workplace bullying is related to more emotional reactions, such as burnout (Nielsen and Einarsen, 2012). Stress is a diagnosis that applies to a collection of responses that occur when individuals oppose a threat to life, whereas emotional exhaustion is a syndrome experienced by long-term exhaustion and reduced attentions in favorite activities. Although it is arguable whether workplace bullying can be seen as the threat of serious injury or loss of life that drives psychological stress and depression (e.g., Mikkelsen and Einarsen, 2002), it is argued that the syndrome found among victims of workplace bullying reflects these negative emotional reactions (Tuckey and Neall, 2014). By enduring bullying and experiencing the increasing strain of trying to manage it, victims are likely to feel exhausted (Lee and Brotheridge, 2006; Tuckey and Neall, 2014). Therefore, this study theorizes the following:

Hypothesis 3: Workplace bullying is positively related to emotional exhaustion.

### Emotional Exhaustion in the Mediating Role Between Workplace Bullying and Voice Behavior

Employee voice is a behavior that is outside an employee’s regular role and likely to be affected by serious issues within the organization; it is influenced by the different levels of emotional reaction among individuals (Seibert et al., 2001). The more exhausted individuals are by their emotional reactions, the more likely it is that they will use less voice. Employees who take the initiative to voice suggestions can promote opportunities for employee participation, benefit from collective thinking, and increase the decision quality of organizations (Weiss and Morrison, 2019). However, when employees who experience workplace bullying are unable to diminish their emotional exhaustion, they may be less likely to engage in voice behavior beyond their main obligations. If employees experience workplace bullying and an exhausted emotional reaction, they may disengage or remove themselves as a way to use voice. Specifically, in reaction to bullying, targets may focus on avoiding the workplace bullying situation and be too exhausted to provide constructive suggestions. This perspective is consistent with the findings of prior research (Sonnentag et al., 2010) that employees who were exhausted by their jobs became uninvolved in work behaviors. Based on AET, it has also been proposed that when individuals are unable to cope with the experience of bullying, workplace bullying directly and indirectly influences their affect, emotions, and behaviors (Glasø et al., 2011). Therefore, this study theorizes the following:

Hypothesis 4: Emotional exhaustion mediates the relationship between workplace bullying and employee voice.

### Emotional Exhaustion in the Mediating Role Between Workplace Bullying and Physical Health

Workplace bullying is related to despondency, compulsive behavior, and social isolation. Prior studies concluded that bullying can be seen as cognitive activation producing emotional reactions (e.g., nausea and burnout; Eriksen and Ursin, 2004; Nielsen and Einarsen, 2012). Cognitive activation or a negative situation that individuals continually experience over a cycle of time based on the negative actions of other people is related to emotional exhaustion and depression (Nielsen and Einarsen, 2012). It has been found that emotional exhaustion, which is produced among bullied targets, is clearly associated with physical health issues (Hoel et al., 2004). Prior research examining the individual experience of bullying has examined that it may generate physical health issues (Einarsen, 2000). The above studies are based on victims’ own assessment of the influence of their experience on their health. Therefore, this study theorizes the following:

Hypothesis 5: Emotional exhaustion mediates the relationship between workplace bullying and physical health.

## Materials and Methods

Before the participants of this study were asked to complete the survey packets, a research ethics sanction was granted by the Human Research Ethics Committee at National Cheng Kung University in Taiwan (No. 107–385). The participants’ responses were kept confidential. The survey packets of this study included questions on workplace bullying, emotional exhaustion, employee voice, physical health issues, and control variables, i.e., participants’ gender, age, education, number of children living at home, and work hours per week. All participants of this study voluntarily joined this study, and their responses were kept confidential. In addition, this study used the Mandarin version of the survey translated from the English version of the survey by native English-speaking researchers before the participants were asked to complete the surveys. The participants of this study were asked to fill in the Mandarin version of the survey.

### Participants and Procedures

The author took steps to avoid common method variance (CMV), in which “variance is attributable to the measurement method rather than to the constructs the measures represent” (Podsakoff et al., 2003, p. 879). Therefore, this study involved two waves of measurements to duplicate the mediating effects of emotional exhaustion in the relationship between workplace bullying and its outcomes, i.e., employee voice and physical health. At Time 1, the author asked the participants to complete measures of demographic characteristics, workplace bullying, and emotional exhaustion. Four weeks later, at Time 2, participants were asked to complete a survey packet on employee voice and physical health.

This study collected survey data from 1,200 participants who worked in a large Taiwanese retail organization. These participants of this study consisted of full-time employees including managers and employees from different divisions in this retail organization. Through personal connections, this corporation’s human resource (HR) manager was contacted to assist and prepare a list of randomly selected employees. The author asked the participants to complete the survey questionnaire after they had finished the employee training program in the organization at Time 1. After 3 months at Time 1, the author asked the participants to complete the second questionnaire in their organizations and provided gifts (worth approximately US$5) to the employees. At Time 1, the author received 1,008 usable surveys back (return rate of 84%). Three months later, at Time 2, the author received 752 surveys back (return rate of 75%). Some personal information of the participants, such as ID numbers and birthdates, was required to assist in matching the different surveys. After unavailable and missing data were eliminated, a total 694 surveys were used in this study. The usable surveys, based on 694 participants, included 525 male employees (75%), whose average age was 41 years (*SD* = 10.81), and 169 female employees (25%), whose average age was 34 years (*SD* = 13.53). The average number of work hours per week was 40 h (*SD* = 20.54).

### Measures

According to suggestion of prior research (Brislin, 1980), native researchers analyzed the survey scales by using a back-translation procedure to confirm equivalence between the Mandarin and English versions. Before the participants of this study completed the Mandarin version of all surveys, therefore, the English version was translated into a Mandarin version. A 5-point Likert scale with responses ranging from never (1) to always (5) in all surveys was used in this study.

### Workplace Bullying

This study assessed workplace bullying using the 22-item scale from the Negative Acts Questionnaire (NAQ; Einarsen and Raknes, 1997) at Time 1. Sample items included “ridicule or insulting teasing” and “being ignored or excluded.” When answering the items, employees were asked to consider their experience of bullying over the past 6 months. The internal consistency estimates were0.93 for work-related bullying.

### Emotional Exhaustion

To assess the employees’ level of emotional exhaustion at Time 1, the study used the Maslach Burnout Inventory-General Survey developed by Schaufeli et al. (1996). The participants assessed their feelings of physical and psychological fatigue and exhaustion on a 5-item scale, with items such as “I feel burned out from my work.” The Cronbach alpha for this scale was 0.86.

### Employee Voice

A six-item scale developed by Van Dyne and LePine (1998) was employed to assess the level of voice behavior at Time 2. The scale assessed the degree to which employees attempted to improve work processes and work conditions and to which they provided constructive suggestions to their organizations. Sample items included “I am willing to propose suggestions at work to affect the department’s future development” and “I take initiative in voicing and encouraging colleagues to participate and affect change for the department’s future development.” The Cronbach α value for the scale was 0.95.

### Physical Health

This scale of perceived health developed by Bookwala (2005) was used to assess participants’ current health at Time 2. This survey used a global single-item with a 5-point scale from the worst possible health to 5 the best possible health. The Cronbach α value for the scale was 0.92.

### Control Variables

The demographic characteristics (i.e., participants’ gender, age, education, number of children living at home, work hours per week, and job position) that are frequently employed in psychological strain research to reduce false results (Bolger et al., 1989; Liu et al., 2008) were controlled in this study. In addition, prior studies have shown that gender (males and females), age, and work hours are likely to affect voice behavior (e.g., Huang et al., 2018; Weiss and Morrison, 2019). This study also controlled for different demographics (e.g., education, number of children, and work hours), which is described by Hoel et al. (2004) to reduce generalizability issues. In addition, this study used open-ended items for age and number of children.

## Results

### Data Statistical Analyses

To examine all data of this study, the author examined all variables, i.e., workplace bullying, emotional exhaustion, employee voice, and physical health, at the individual level and used LISREL 8.8 (Jöreskog and Sörbom, 2006) to measure the constructs’ distinctiveness and evaluate the various fit indices for confirmatory factor analysis (CFA). Prior research recommend that fit indices be used to determine model fit: chi-square test, root mean square error of approximation (RMSEA; = 0.05), comparative fit index (CFI; = 0.90), non-normed fit index (NNFI; = 0.90), standardized root mean square residual (SRMR; = 0.05), and adjusted goodness-of-fit index (AGFI; = 0.90). The hypothesized mediation model was examined by means of the Sobel test and the bootstrapping approach, which are statistical procedures used to calculate effect sizes and hypothesis models for mediation effects (Preacher and Hayes, 2008).

### Analyses of Measurement Models

This study utilized Harman’s single-factor test, which was executed by including all items of the study in a fixed one-factor unrotated factor analysis to examine the effects of CMV among the variables (Podsakoff et al., 2003; Fuller et al., 2016). Given a total explained variance of 44.49%, this study did not find any evidence of CMV. In addition, the author took several steps to test the influence of CMV, which each measurement model could load on a latent CMV factor and on its underlying theoretical construct. In Table 1, a four-factor structure with workplace bullying, emotional exhaustion, employee voice, and physical health fit the data better [χ^2^(694) = 682.51 (*p* < 0.01); *df* = 124; model Akaike information criterion (AIC) = 8,786.51; RMSEA = 0.06; NNFI = 0.90; SRMR = 0.05; CFI = 0.93; and AGFI = 0.91], which allowed to test the hypotheses. The findings of the model fit indices in Table 1 show that the baseline model showed considerably better fit than alternative Models 1, 2, and 3. Based on the findings of alternative models in Table 1, the delta chi-squares (Δχ^2^; Model 1 = 838.38; Model 2 = 948.52; Model 3 = 1,456.12) and model AIC (Model 1 = 10,536.38; Model 2 = 10,442.52; Model 3 = 14,748.12) were statistically significant. In addition, for each construct, the average variance extracted (AVE) was larger than 0.50 (workplace bullying = 0.58; emotional exhaustion = 0.84; employee voice = 0.51; physical health = 0.52), which suggested that discriminant validity was acceptable. All factor loadings in the baseline model of this study were standardized loadings ranging from 0.52 to 0.92 and were significant based on the evaluation of factor loading and factor covariance. It showed support for convergent validity.

**TABLE 1:** Comparison of measurement models.

**Model**	**Factors**	**χ^2^**	**Model AIC**	***df***	**Δχ^2^a**	**RMSEA**	**NNFI**	**CFI**	**SRMR**	**AGFI**
Baseline model	Four factors	682.51**	8,786.51	224	-	0.06	0.90	0.93	0.05	0.91
Model 1	Three factors: Two outcomes were combined into one factor, one mediator and one independent variable	838.38*	10,536.38	227	155.87**	0.08	0.73	0.77	0.09	0.81
Model 2	Two factors: Two outcomes and one mediator were combined into one factor, and one independent variable	948.52*	10,442.52	229	266.05**	0.15	0.50	0.54	0.14	0.72
Model 3	One factor: All four factors were combined into one factor.	1,456.12*	14,748.12	130	773.61**	0.30	0.52	0.57	0.17	0.62

AIC, Akaike information criterion; RMSEA, root mean square error of approximation; NNFI, non-normed fit index; CFI, comparative fit index; SRMR, standardized root mean square residual; AGFI, adjusted goodness-of-fit index.

*p < 0.05; **p < 0.01.

*^a^Baseline model was compared with Models 1–3.*

### Hypothesis Testing

The means, standard deviations, and intercorrelations among all variables were calculated by an SPSS (Preacher and Hayes, 2008) before examining the hypotheses of this study. This study predicted a positive effect of workplace bullying on employee voice in Hypothesis 1. The results of this study show a significant relationship between workplace bullying and employee voice (*r* = 0.25, *p* < 0.01; see Table 2). Hypothesis 2 predicted a negative effect of workplace bullying on physical health. The findings of this study show a significant relationship between workplace bullying and physical health (*r* = −0.21, *p* < 0.01) in Table 2. In addition, there was a positive relationship between workplace bullying and emotional exhaustion (*r* = 0.44, *p* < 0.01).

**TABLE 2:** Means, standard deviations, Cronbach’s alpha, and intercorrelations among the study.

**Variable**	***M***	***SD***	**1**	**2**	**3**	**4**	**5**	**6**	**7**	**8**	**9**	**10**
1. Gender	1.24	0.43	(−)									
2. Age	39.47	11.92	−0.26**	(−)								
3. Number of children	1.68	0.86	−0.05	0.21**	(−)							
4. Education	2.53	0.88	−0.18**	0.19**	−0.06	(−)						
5. Work hours	29.99	20.54	−0.31**	0.51*	0.08*	0.35**	(−)					
6. Job position	1.08	0.30	−0.01	0.04	0.02	0.06	0.05	(−)				
7. Workplace bullying	1.87	0.93	0.15**	−0.10*	−0.09*	−0.02	−0.03	−0.02	(0.93)			
8. Emotional exhaustion	2.99	0.90	−0.04	0.11**	−0.04	0.10**	0.28**	−0.04	0.44**	(0.86)		
9. Employee voice	3.38	0.71	0.16**	−0.18**	−0.03	−0.07	−0.20**	−0.03	0.25**	−0.06	(0.95)	
10. Physical health	3.05	1.10	0.10**	−0.18**	−0.03	−0.07	−0.41*	0.02	−0.21**	−0.41**	0.07	(0.92)

Coefficient alphas are in parentheses on the diagonal.

*p < 0.05; **p < 0.01.

**FIGURE 1:**
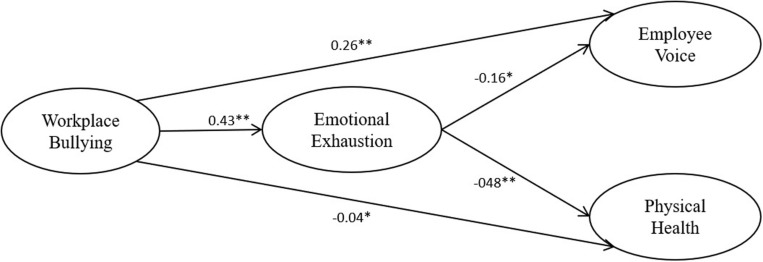
Summary of results. Results are obtained from the structural equation modeling (SEM). *p < 0.05; **p < 0.01.

This study used LISREL 8.8 (Kline, 2011) to examine the hypothesized structural equation modeling (SEM) and employ weighted least-square estimators to evaluate the theoretical model, which had two partial mediation models as shown in Table 3. Compared with the three alternative models of this study in Table 3, the theoretical model of this study predicted two paths: workplace bullying would be related to employee voice through emotional exhaustion and to physical health through emotional exhaustion. The theoretical model showed the optimum fit [χ^2^(694) = 2.31, *df* = 1; RMSEA = 0.04; CFI = 0.99, and AGFI = 0.98]. These findings of this study suggest that the theoretical model best fits the data based on the principle of model parsimony. In addition, Figure 1 shows that workplace bullying was significantly related to employee voice (β = 0.26; *p* < 0.01), supporting Hypothesis 1, and shows a negative effect of workplace bullying on physical health (β = −0.04; *p* < 0.05), supporting Hypothesis 2. In addition, there was a positive relationship between workplace bullying and emotional exhaustion (β = 0.43; *p* < 0.01) presented in Figure 1, supporting Hypothesis 3. This study conducted a bootstrapping approach (Preacher and Hayes, 2008) to examine the indirect effect of workplace bullying on employee voice and physical health through emotional exhaustion. The findings of this study showed that the indirect effect of workplace bullying on employee voice through emotional exhaustion was a significant (*z* = −4.82, *p* < 0.01, with a 99% bootstrap confidence interval ranging from -0.12 to -0.03) and found that the indirect effect from workplace bullying on physical health via emotional exhaustion was significant (*z* = −7.92, *p* < 0.01, with a 99% bootstrap confidence interval ranging from -0.30 to -0.14), supporting Hypotheses 4 and 5.

**TABLE 3:** Comparison of structural equation models for employee’s psychological strain.

**Model**	**χ^2^**	***df***	**Δχ^2^**	**RMSEA**	**CFI**	**AGFI**
Theoretical model	2.31**	1	−	0.04	0.99	0.98
Alternative Model 1	66.00**	3	63.69**	0.17	0.79	0.85
Alternative Model 2	5.89**	2	3.58*	0.06	0.98	0.97
Alternative Model 3	65.87**	2	63.56**	0.22	0.79	0.77

Alternative Model 1: two full mediations. Alternative Models 2 and 3: one partial mediation and one full mediation.RMSEA, root mean square error of approximation; CFI, comparative fit index; AGFI, adjusted goodness-of-fit index.*p < 0.05; **p < 0.01.

## Discussion

This study explored how workplace bullying leads to employee voice and damages physical health through emotional exhaustion by using two-wave data collected in a large Taiwanese retail organization. The findings of this study revealed that (1) workplace bullying was positively related to employee voice and damaged physical health, (2) the significant relationship between workplace bullying and employee voice was partially mediated by emotional exhaustion, and (3) emotional exhaustion partially mediated the relationship between workplace bullying and physical health. These findings imply that when workplace bullying occurs, victims recklessly speak up to alter the bullying situation and their physical health decreases.

This study suggests that employees who are bullied in the workplace are more likely to challenge the *status quo* by presenting opinions on workplace issues (Weiss and Morrison, 2019). Voice behavior can enhance the victim’s status in an organization and impact interpersonal relationships because it implies that the victim is willing to address threats on behalf of others. Voice expressions may vocalize ways to resolve problems with work situations in which individuals experience unfair treatment in the workplace (Maynes and Podsakoff, 2014). Additionally, workplace bullying appears to harm health. Consistent with Hoel et al. (2004), experiences of social isolation, verbal hostility, and physical threats among bullied employees in the workplace are manifested in physical health issues. The victims reported nervous symptoms, sweating, and insomnia when workplace bullying exists. Indeed, the findings of this study indicate that under workplace bullying, victims display physical health issues.

### Theoretical Implications

The first main theoretical contribution of this study is its exploration of how workplace bullying leads to employee voice, thereby developing one explanation for why victims speak up to challenge the *status quo* by presenting opinions. The findings of this study are not consistent with prior studies (Rai and Agarwal, 2018, 2019) that employees engage in silence to avoid the risk of personal resource loss and opportunities when workplace bullying occurs. Voice represents the voluntary initiation of beneficial changes to improve the situation in an organization (e.g., Grant et al., 2009), and this study provides a rationale for this behavior: victims express their opinions to resolve problems with negative work situations. Essentially, this study investigated whether employee voice is stimulated by workplace bullying. In conjunction with the damage from bullying, victims may be able to resolve social isolation and harm to their physical health in the workplace. As a bullying situation escalates, bullied employees are likely to find their inner strength enhanced, so that they can voice their own opinions regarding the negative *status quo* to improve job security. These effects imply a psychological decision by bullied employees to respond to their workplace situation to employers and organizations.

The second contribution developed from finding support for emotional exhaustion as a critical mediator of the affective event process. This study examined the connections between workplace bullying and its outcomes, i.e., employee voice and physical health, and found that the mediation of emotional exhaustion between workplace bullying and its outcomes best fits the data. Although few longitudinal studies have tested the relationship between workplace bullying and its outcomes (e.g., Park and Ono, 2017), the findings of this study are consistent with those of Tuckey and Neall’s (2014) study, which supported the link from workplace bullying to emotional exhaustion. Additionally, this study finds support for the mediating role of emotional exhaustion in the relationship between workplace bullying and employee voice and in the link between workplace bullying and physical health.

The final contribution of this study stems from its finding that workplace bullying is an ongoing problem in organizations. This study observed that bullying experience exists significantly among individuals across a timeframe of 1 month. Prior bullying studies have used a cross-sectional design (e.g., Neall and Tuckey, 2014), while this study provides a time-lag design of 4 weeks of research to explore the effects of workplace bullying on its outcomes, including behavioral and physical issues. Bullying experiences may directly and indirectly target producing employee voice and damaging physical health. All of these probabilities emphasize the importance of clearly examining the link between workplace bullying and emotional exhaustion.

### Practical Implications

Workplace bullying is a reality in organizations. It is important to recognize that the damaging health conditions and negative behaviors reported by many victims of bullying are an effect of their bullying experience. The findings of this study may help HR managers and employers to understand the effects of workplace bullying. This study also suggests that employers and HR managers should recognize that workplace bullying may lead victims to speak up about their negative experience and exhaust their emotional reactions. The threat of workplace bullying may influence emotional and physical health issues. Employers and managers must publicly create policies such as “managing with respect” and “zero tolerance for bullying at work.” Specifically, victims of bullying may not be considered good colleagues because they may be emotionally exhausted by the bullying situation. Thus, employers should encourage employees to create positive social relations with colleagues to avoid workplace bullying.

Workplace bullying is a complicated experience at the individual level. The results of this study suggest that early personal assistance and involvement should help employees avoid becoming systematically and organizationally harassed and can reduce emotional and physical damage. Particularly, employers and managers have to protect the bullied victims and help them join in social relations with colleagues. Prior studies have found that encouraging employees to understand workplace bullying, openly imposed policy, e.g., zero tolerance for workplace bullying, and clear processes for informing workplace bullying is valid to avoid bullying at work (Tuckey and Neall, 2014; Rai and Agarwal, 2018). Hopefully, the findings of this study can provide new perceptions to influence different opinions on the AET process.

### Limitations and Future Research

This study has several limitations that should be addressed. First, there were fewer participants in this study than in similar studies. The results of this study may not be generalized to all employees in other countries even if the findings of this study found that workplace bullying was positively related to employee voice and damaged physical health, and emotional exhaustion was an important mechanism between workplace bullying and its consequences in this research. Future research investigating whether these prototypes differ in other countries would be valuable and may examine diverse cultural samples to reduce generalizability issues. Second, it may be that participants did not respond accurately because the questionnaire addressed sensitive issues that could lead victims to worry about increased exposure to workplace bullying. Indeed, workplace bullying is a crucial issue that may diminish social interactions with colleagues in the organization. Colleagues may not assist victims of workplace bullying because they do not want to be another victim. Future research should seek to help researchers explain the details of the survey and research to avoid false and inaccurate responses. Third, this study shows that workplace bullying can influence negative reactions and outcomes in organizations through affective processes based on AET (Weiss and Cropanzano, 1996). However, a possible reverse relationship between workplace bullying and its consequences at Time 1 and Time 2 is possible even if this study conducted a two-time period design. All study variables may be influenced by an unobserved variable and create any causal claims. It is important to develop strong theory in behavioral sciences. Fourth, this study did not consider the relationship between employee voice and physical health. When employees strive to make valuable suggestions to organizations, a negative physical condition may occur to some degree. For future research, this relation of employee voice and physical health may be examined because employees proactively engaging in voice behavior may influence their health status. Finally, even though data were collected from employees of a large Taiwanese retail organization, this study might not be applicable to general issues in different organizations because of practical constraints; in the future, researchers could select multiple organizations to avoid the unique characteristic of one organization.

## Conclusion

In conclusion, this study explains the mediating role of emotional exhaustion in the relationship between workplace bullying and employee voice and in the link between workplace bullying and physical health. The findings of this study support the notion that the bullied victims react effectively to voice because workplace bullying should be improved to standard social relationships in the organization. In addition, the results of this study find that under workplace bullying, victims display physical health issues. All in all, this study has provided some new directions (i.e., workplace bullying process and its outcomes, i.e., voice behavior and physical issues) by drawing on AET.

## Data Availability Statement

The raw data supporting the conclusions of this article will be made available by the author, without undue reservation, to any qualified researcher.

## Ethics Statement

The ethics sanction of this study was granted by the Human Research Ethics Committee at National Cheng Kung University in Taiwan (No. 107-385). The participants provided their written informed consent to participate in this study.

## Author Contributions

The author confirms being the sole contributor of this work and approved it for publication.

## Conflict of Interest

The authors declare that the research was conducted in the absence of any commercial or financial relationships that could be construed as a potential conflict of interest.
